# Ectopic expression of *AtDGAT1*, encoding diacylglycerol *O*-acyltransferase exclusively committed to TAG biosynthesis, enhances oil accumulation in seeds and leaves of Jatropha

**DOI:** 10.1186/s13068-016-0642-7

**Published:** 2016-10-21

**Authors:** Devendra Kumar Maravi, Sanjeev Kumar, Prabin Kumar Sharma, Yasufumi Kobayashi, Vaibhav V. Goud, Nozomu Sakurai, Hiroyuki Koyama, Lingaraj Sahoo

**Affiliations:** 1 Center for Energy, Indian Institute of Technology Guwahati, Guwahati, 781039 India; 2 Department of Biosciences and Bioengineering, Indian Institute of Technology Guwahati, Guwahati, 781039 India; 3 Applied Biological Sciences, Gifu University, 1-1, Yanagido, Gifu, 501-1193 Japan; 4 Department of Chemical Engineering, Indian Institute of Technology Guwahati, Guwahati, 781039 India; 5 Department of Technology Development, Kazusa DNA Research Institute, 2-6-7 Kazusa-Kamatari, Kisarazu, Chiba 292-0818 Japan

**Keywords:** AtDGAT1, Biodiesel, Jatropha, Leaf, Seed oil, Transgenic, Triacylglycerols (TAGs)

## Abstract

**Background:**

*Jatropha curcas* is an important biofuel crop due to the presence of high amount of oil in its seeds suitable for biodiesel production. Triacylglycerols (TAGs) are the most abundant form of storage oil in plants. Diacylglycerol *O*-acyltransferase (DGAT1) enzyme is responsible for the last and only committed step in seed TAG biosynthesis. Direct upregulation of TAG biosynthesis in seeds and vegetative tissues through overexpression of the *DGAT1* could enhance the energy density of the biomass, making significant impact on biofuel production.

**Results:**

The enzyme diacylglycerol *O*-acyltransferase is the rate-limiting enzyme responsible for the TAG biosynthesis in seeds. We generated transgenic Jatropha ectopically expressing an *Arabidopsis DGAT1* gene through *Agrobacterium*-mediated transformation. The resulting *AtDGAT1* transgenic plants showed a dramatic increase in lipid content by 1.5- to 2 fold in leaves and 20–30 % in seeds, and an overall increase in TAG and DAG, and lower free fatty acid (FFA) levels compared to the wild-type plants. The increase in oil content in transgenic plants is accompanied with increase in average plant height, seeds per tree, average 100-seed weight, and seed length and breadth. The enhanced TAG accumulation in transgenic plants had no penalty on the growth rates, growth patterns, leaf number, and leaf size of plants.

**Conclusions:**

In this study, we produced transgenic Jatropha ectopically expressing *AtDGAT1*. We successfully increased the oil content by 20–30 % in seeds and 1.5- to 2.0-fold in leaves of Jatropha through genetic engineering. Transgenic plants had reduced FFA content compared with control plants. Our strategy of increasing energy density by enhancing oil accumulation in both seeds and leaves in Jatropha would make it economically more sustainable for biofuel production.

## Background

The diminishing fossil fuel stock and soaring international crude oil price have renewed the interest in alternative sources of fuels. Oils from oilseed crops that are largely in the form of triacylglycerols (TAG) are the promising source of renewable supply of fuels in the form of biodiesel [1, 2]. *Jatropha curcas* L. is an important non-edible oilseed crop which received worldwide attention as a biodiesel feedstock. It is a shrub grown in tropical and subtropical regions of the world. The seeds which contain 30–42 % of oil can be directly blended with diesel or transesterified for use as biodiesel. In addition to high oil content, favorable oil composition for biodiesel such as seed oil with approximately 75 % unsaturated fatty acids (FAs) [3, 4], and a high level (around 47 %) of linoleic acid (C18:2) [5], Jatropha plants have a short gestation period, easy adaptation to various agroclimatic conditions [6, 7], and ability to grow on marginal and semi-marginal lands, making this plant the most sought oilseed crop among the non-edible oil-yielding crops for biodiesel production [8].

Despite the significance of Jatropha seed oil as a potential source for biodiesel, not much research efforts have been made through breeding or transgenic approaches to improve its seed oil content and quality for sustainable biodiesel production. Transgenic approaches offer immense opportunities to improve oil content and quality through manipulation of oil biosynthetic pathway in both seed and leaves [9–11].

TAGs, which consist of three FA chains (usually C16 or C18) covalently linked to glycerol, serve as an energy reserve for the seed germination, and seedling growth and development. Depending on the source of plants, TAGs may contain FAs with different chain lengths and extent of saturation, and diverse modified FAs [10]. Plant TAGs are generally stored in small organelles, oil bodies, which are assembled in the developing seeds, flower petals, pollen grains, and fruits of a large number of plant species [12, 13]. A series of condensation, reduction, and dehydration reactions led to fatty acid synthesis in plastid, and the free fatty acids (FFAs) are transported to endoplasmic reticulum (ER). FFAs are then involved in sequential acylation of the sn-1, sn-2, and sn-3 positions of glycerol-3-phosphate with acyl-CoA to finally yield TAG through Kennedy pathway.

In the Kennedy pathway, diacylglycerol acyltransferase (DGAT), which catalyzes the terminal step, is the only enzyme that is exclusively committed to TAG biosynthesis using acyl-CoA as its acyl donor [14] and plays a vital role in diverting fatty acid flux towards the formation of TAGs [15, 16]. Two different DGAT gene family members, DGAT1 and DGAT2 that differ considerably in sequence, have been attributed to have a non-redundant role in TAG biosynthesis [17, 18]. However, the preferences for either of these two forms for TAG production and its accumulation during seed development have been found to be species specific [19]. Ever since *DGAT1* gene from *Arabidopsis* was identified simultaneously by three laboratories [20–22], its homologues were subsequently reported from several other plants including tobacco [22], canola [23], castor bean [24], burning bush [25], soybean [26], peanut [27], tung tree [18], *Tropaeolum majus* [13], *J. curcas* [28–30], *Populus trichocarpa* [31], and Indian mustard [32]. Therefore, manipulation of DGAT1 gene expression has a significant effect on the improvement of the oil content and alteration of the fatty acid composition. *Arabidopsis* lines lacking DGAT1 were found deficient in DGAT activity and accumulated less oil with decreased TAG/DAG ratios [20, 21, 33, 34], while RNAi suppression of DGAT1 in tobacco resulted in decreased seed oil content and an increase in protein and carbohydrate [35]. On the contrary, overexpression of DGAT1 has lead to the increase in levels of oil in *Arabidopsis* [36], *Brassica napus* [32, 37], tobacco [38], soybean [39, 40], castor [41], maize [42, 43], and Indian mustard [32]. Although several genetic transformation methods have been reported for *J. curcas* [44–46], this is the first report of using genetic engineering approach to improve its oil quantity and quality in seeds and leaves.

In the present study, we demonstrate the constitutive overexpression of *AtDGAT1* results in the enhanced accumulation of TAGs and better oil attributes, in both seeds and leaves of transgenic Jatropha without compromising the seed yield, and morphological and developmental features.

## Results

### Generation of *AtDGAT1* overexpressing transgenic Jatropha using a constitutive promoter and molecular characterization

**Fig. 1:**
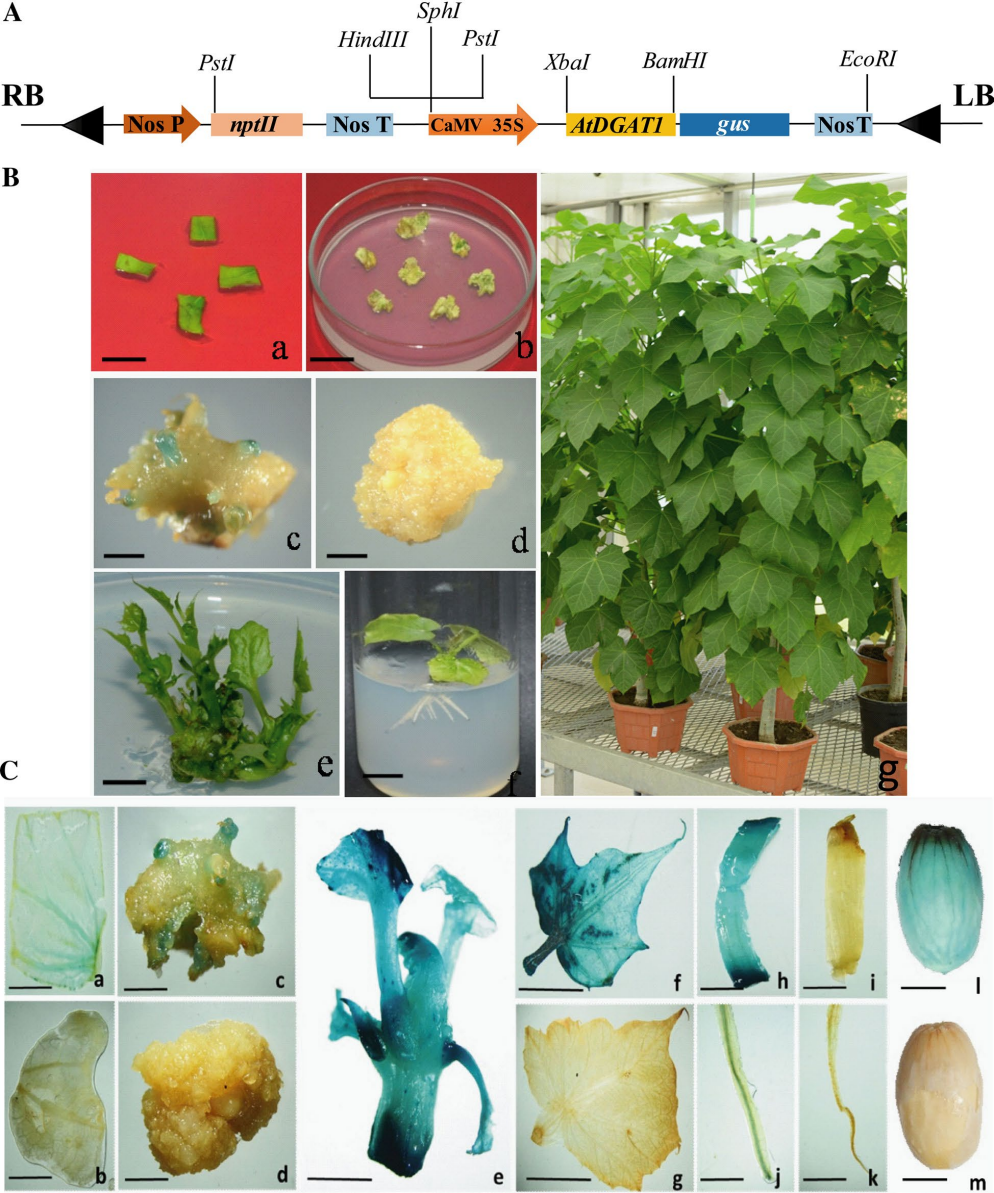
Development of transgenic *Jatropha curcas* plants expressing *AtDGAT1*. **A** T-DNA region (6.2 kb) of pBI121CaMV35S::*AtDGAT* plasmid. *RB* right border, *LB* left border, *CaMV 35S promoter Nos P* nopaline synthase promoter, *Nos T* nopaline synthase terminator, *NptII* neomycin phosphotransferase, ß-glucuronidase. **B** *Agrobacterium*-mediated genetic transformation of *Jatropha curcas* with 35S::*AtDGAT* construct: *a* excised cotyledonary leaf explants subjected to *Agrobacterium tumefaciens*-mediated transformation and cultured on kanamycin-free callus induction medium (*bar* 5 mm); *b* formation of callus from explant (*bar* 8 mm); *c* Stable GUS expression in buds induced from transformed callus (*bar* 8 mm); *d* untransformed control callus (*bar* 8 mm); *e* elongated putative transformed shoots on kanamycin selection (*bar* 1 cm); *f* rooted transformed plantlet (*bar*  1 cm); *g* acclimatized transformed plant. **C** Stable *gus* expression in transgenic plant tissues. *a* transformed cotyledonary leaf segment; *b* untransformed leaf segment; *c* emerging shoots from callus showing *gus* expression; *d* callus from untransformed tissues; *e gus* expression in in vitro shoot; *f gus* expression in leaf; *g* control leaf; *h gus* expression in stem; *i* control stem; *j* gus expression in transgenic root and *k* control root; *l gus* expression in transgenic seeds and *m*, control seed. (*Bar* 0.5 cm)

**Fig. 2:**
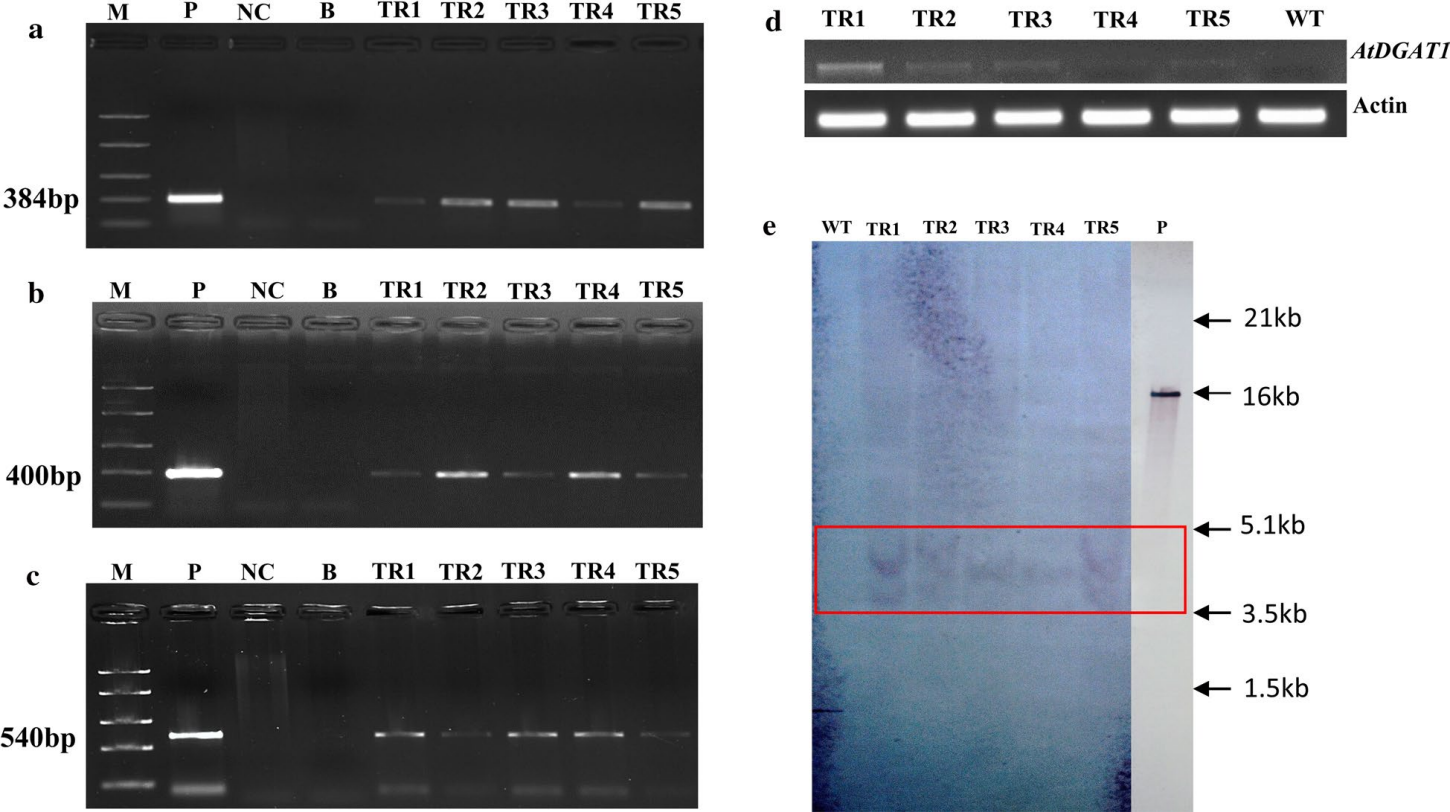
Molecular analysis of transgenic plants of *Jatropha curcas*. **a** PCR amplification of the 384-bp fragment of *AtDGAT* gene; **b** PCR amplification of the 400-bp fragment of *gus* gene; **c** PCR amplification of the 540-bp fragment of *nptII* gene. *Lane* M molecular marker, *Lane* TR1, TR2, TR3, TR4, TR5, genomic DNA from five transgenic plant *Lane* P, pBI121::*AtDGAT1* plasmid (positive control), *Lane* NC, DNA from untransformed plant (negative control) and *Lane* B, blank; **d** Transcript abundance of *AtDGAT1* in transgenic line of *J. curcas*. Expression analysis was carried out by semi-quantitative PCR using *actin* as an internal control. **e** Southern blot analysis of transgenic plants expressing *AtDGAT1*,the plasmid and 60 µg genomic DNA was digested with *BamHI*, and hybridized with PCR-amplified *nptII* probe, lane WT genomic DNA from untransformed plant, *Lane* TR1–TR5 genomic DNA from transgenic lines, *Lane* P *BamHI* digested pBI121::*AtDGAT1* plasmid

To investigate the impact of constitutive overexpression of *Arabidopsis DGAT1* cDNA on TAG accumulation in leaves and seeds of Jatropha, we prepared a 35S::AtDGAT1 construct that consisted of *AtDGAT1* fused to *gus* reporter gene driven by CaMV35S promoter and *nptII* as plant selection marker (Fig. 1A). We generated transgenic Jatropha plants harboring *AtDGAT1* through *Agrobacterium*-mediated transformation of cotyledonary leaf segment explants and selection of transformed shoots through kanamycin-based selection. Putative transformed shoots were rooted on kanamycin-free rooting medium and successfully hardened and acclimatized in greenhouse (Fig. 1B, a–g). Stable GUS expression was checked randomly in the cultures, at various developmental stages for validating the strength of kanamycin selection in recovery of transgenic plants. Stable GUS expression was detected in transformed cotyledonary leaf segment explants, developing shoot buds from callus, and regenerated shoots, leaf, stem, roots, and seeds of transgenic plants (Fig. 1C, a–m). These plants were confirmed by genotyping through genomic DNA PCR using primers specific to *AtDGAT1*, *nptII*, and *gus*. Amplification of 384-bp fragment corresponding to the *AtDGAT1*, a 540-bp fragment internal to *nptII*, and 400-bp internal to *gus* confirmed the transgenic plants (Fig. 2a–c).

We randomly selected five *AtDGAT1* Jatropha transgenic lines and extracted RNA for expression analysis. Semi-quantitative RT-PCR analysis revealed abundance of AtDGAT1 mRNA in transgenic lines, albeit high expression in transgenic lines TR1, TR2, and TR3, and moderate expression in TR4 and TR5 in contrast to the wild type (Fig. 2d). We generated several independent lines expressing *AtDGAT1* in which the growth rates, growth patterns, leaf number, and leaf size were all observed normal as compared to their counterpart wild type.

Five randomly selected PCR-positive independent T_0_ transgenic Jatropha lines recovered on kanamycin selection medium were screened by Southern hybridization to confirm the integration of *nptII* gene using a 0.54 kb *nptII* probe. Two of the transgenic lines (TR3 and TR4) exhibited single copy integration events (Fig. 2e), whereas the three transgenic lines (TR1, TR2, and TR5) showed integration of two copies (Fig. 2e). No hybridization signal was detected in control WT plant (Fig. 2e).

### *AtDGAT1* overexpressing transgenic Jatropha plants accumulated enhanced levels of storage lipids in their leaves and seed kernels

We generated AtDGAT1 transgenic Jatropha plants to see if an overexpression of AtDGAT1 in Jatropha would also lead to increased oil accumulation in leaf biomass and seed kernel. As we intended to specifically allow lipid accumulation in the leaves as well as in mature seeds, the *AtDGAT1* was placed under the control of the constitutive CaMV35S promoter.

**Fig. 3:**
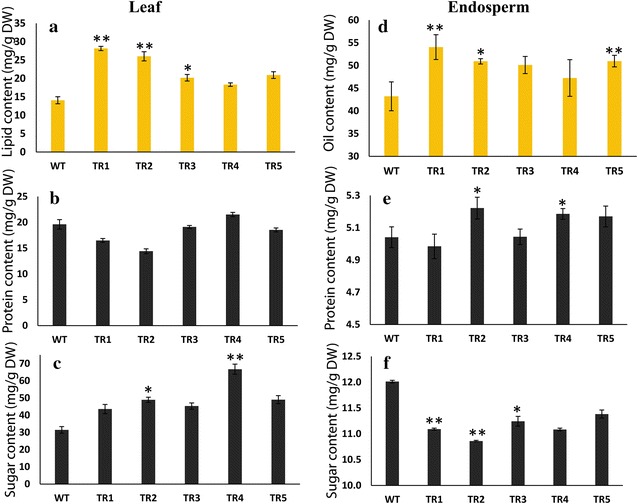
Variation in leaves and seed lipids, protein, and sugar contents. **a** Leaf lipid content; **b** leaf protein content; **c** leaf sugar content analysis, and **d** seed oil; **e** seed protein content; **f** seed sugar content analysis in wild-type and *AtDGAT1*-*Jatropha curcas* transgenic lines. Difference between untransformed (WT) and transgenic lines was significant at *P* < 0.05 (*), *P* < 0.01 (**) by Tukey test. Values are shown as mean ± SD (*n* = 3)

To investigate total lipid content in mature seeds and leaves of transgenic Jatropha plants, we harvested leaves and mature seeds from five individual transgenic plants and separated the seed kernels for further studies. Compared to control (WT) plants, all tested transgenic lines showed significant increase (1.5- to 2-fold) in total lipid content in leaves (Fig. 3a). The leaves of best transgenic line (TR1) accumulated twofold higher total lipid content than the control (WT) plants which represented a 100 % increase of total lipid in transgenic line (TR1). The seed kernels of all the transgenic lines tested showed enhancement of total lipid content by 20–30 % on a relative basis as compared to WT plants (Fig. 3d). The best transgenic line (TR1) showed a 30 % relative increase of total lipid as compared to WT plants (Fig. 3d).

We analyzed the five *AtDGAT1* transgenic Jatropha lines to find out the possible changes in protein and carbohydrate content at the expense of increased lipid accumulation in leaves and seed kernels, as the precursors of fatty acid biosynthesis in plants are derived from sugar during photosynthesis. The protein content in leaves of transgenic lines showed no significant change as compared to WT plants (Fig. 3b). However, seed kernels of transgenic lines showed a minor increase in protein content except in transgenic line TR1 and TR2 that showed an insignificant decrease in protein content as compared to WT (Fig. 3e). It was observed that the level of total soluble sugar in leaves was significantly increased (38–112 %) in transgenic lines tested as compared to leaves of WT plants (Fig. 3c). The transgenic lines had total soluble sugar in the range of 43.60–66.70 mg g^−1^ FW as compared to wild-type 31.45 mg g^−1^ FW (Fig. 3c). These results suggest that increased accumulation of total soluble sugar may have contributed to reallocation of precursor for enhanced TAG synthesis in transgenic leaves [47]. However, the seed kernels of transgenic lines showed a corresponding decrease in sugar content as compared to wild-type plants (Fig. 3f). These observations suggest that the reallocation of precursor of photosynthesis to TAG biosynthesis is more in leaves than seeds of transgenic AtDGAT1 Jatropha lines, and possibly the contribution to TAG biosynthesis in these transgenic lines by leaves is more than seeds which may be due to the lower expression of 35S-driven genes in the seed versus vegetative tissue. In previous studies, it has been found that 35S does not express as highly in *Arabidopsis* seed and germline tissue compared to vegetative tissue like leaves [48, 49].

**Fig. 4:**
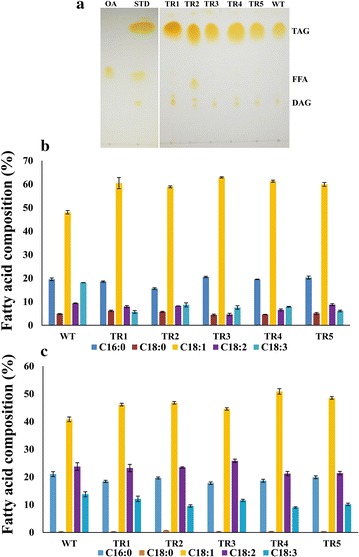
Triacylglycerol accumulation and Fatty acid profiling in transgenic *Jatropha curcas*. **a** TLC separation of neutral lipids from transgenic lines and wild-type *J. curcas*; 150 µg lipids was fractioned by Silica Gel plates, triacylglycerol (TAG), free fatty acids (FFA) and diacylglycerol (DAG), oleic acid (OA), and standard (STD). Fatty acid profiling of transgenic and wild-type *J.* (**b**) leaves; **c** seed kernel

In order to characterize TAG accumulation in mature seed kernels, we used TLC on silica gel plates to analyze qualitatively the total neutral lipids from control (WT) plants and five transgenic lines. The TLC plates revealed an overall increase in TAG content in seeds of all *AtDGAT1* over expressing transgenic Jatropha lines as compared to control (WT) plants (Fig. 4a). Furthermore, the DAG content in seeds of transgenic lines showed a relative increase as compared to WT plants (Fig. 4a). FFA was not detected in seeds of any of the transgenic lines except TR2 (Fig. 4a). All the five transgenic lines showed a very similar level of TAG accumulation in their seeds (Fig. 4a). These results suggest that *AtDGAT1* encodes a functional protein capable for catalyzing the final rate-limiting step of TAG biosynthesis in transgenic Jatropha lines expressing *ATDGAT1*.

To determine and compare the FA profiles between the transgenic lines and WT plants, the fatty acids methyl esters resulting from transesterification of seed oil and leaf lipids were quantified by GC. A significant variation in composition of FAs was detected among the leaves of control and transgenic plants. The leaves of transgenic lines showed a relative increase of oleic acid (18:1) by 20–31 % as compared to WT plants (Fig. 4b). In addition, the level of linolenic acid (18:3) showed a reduction from 18.17 to 5–8 % in transgenic lines as compared to WT plants. There were no marked changes observed in the level of palmitic (16:0), stearic (18:0), and linoleic (18:2) acid in leaves of transgenic lines as compared to WT plants (Fig. 4b). Increased TAG accumulation in seed kernels was accompanied by a profound change in fatty acid composition in TAG fraction (Fig. 4c). GC analysis showed that oleic acid (18:1) accumulation in seed kernels of transgenic lines increased by 9–25 % as compared to WT. On the other hand, the level of linolenic acid (18:3) in seed kernels decreased by 12–35 % in transgenic lines as compared to WT (13.79 %) (Fig. 4c). The linoleic acid (18:2) levels in seed kernels of transgenic lines showed the similar abundance to that of WT, and in contrast, the lines TR4 and TR5 showed the level of linoleic acid decreased by 9.94 and 10.04 %, respectively (Fig. 4c). The level of palmitic acid (16:0) in seed kernels decreased by 12–21 % in all transgenic lines as compared to WT (Fig. 4c). There were no changes in the stearic acid (18:0) levels in seed kernels of the transgenic lines (Fig. 4c).

### *AtDGAT1* overexpressing transgenic Jatropha lines have increased number of oil bodies in leaves

**Fig. 5:**
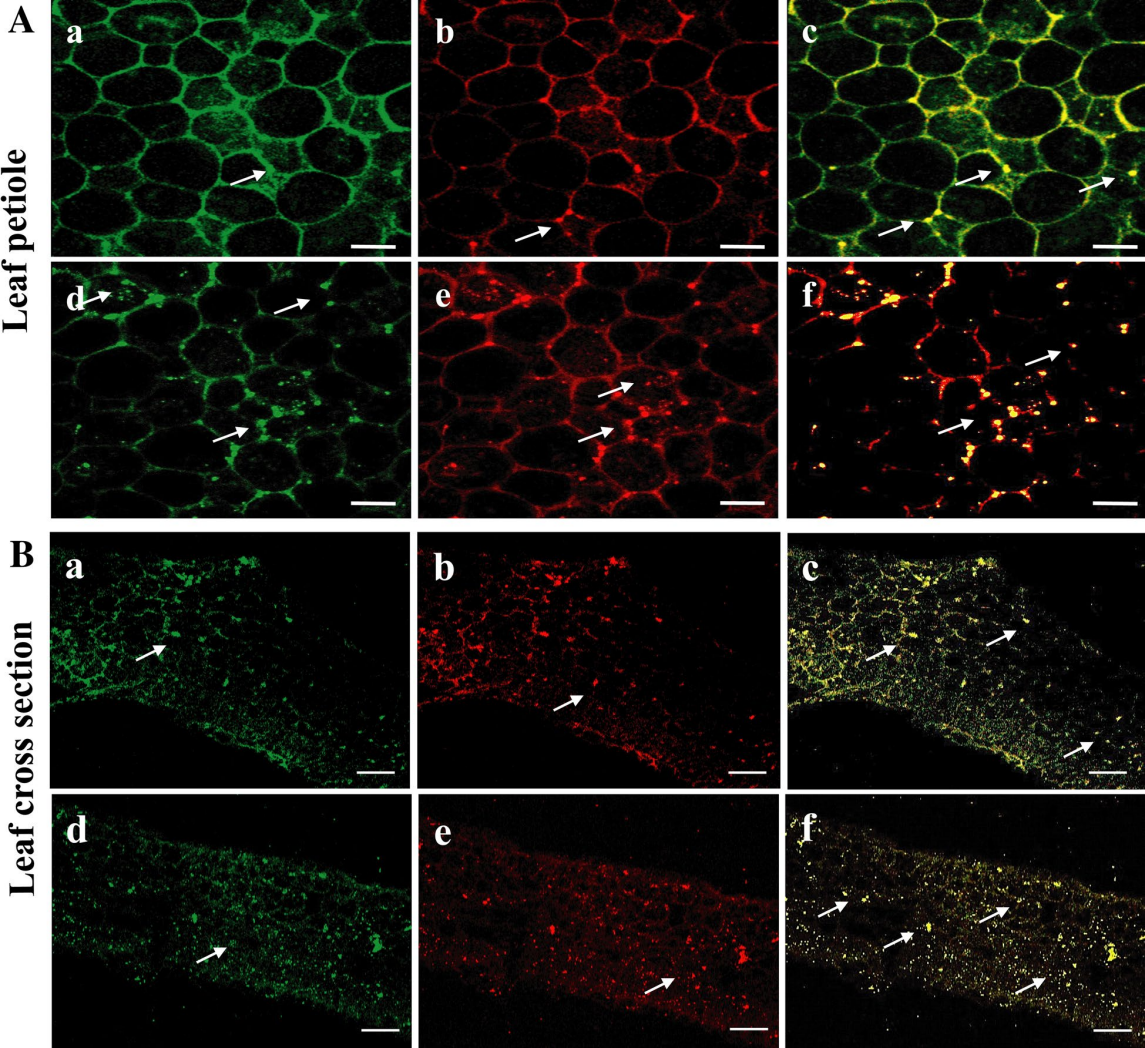
Oil droplets are abundant in leaf sections of *AtDAGT1* overexpressing transgenic lines. Confocal fluorescence image of (**A**) leaf petiole of wild type (*a*–*c*) and transgenic lines (*d*, *e*) showing oil droplets (*arrow*) and **B** leaf cross-section of wild type (*a*–*c*); transgenic line (*d*–*f*) stained with Nile *red*. *Green* and *red* channel is used for imaging, merged images showing *golden-yellow dots* of oil droplets. *Bar* 20 µm

TAGs, the predominant plant storage neutral lipids with twice the energy density of cellulose, are used to generate biodiesel [50]. Increased demand to produce more energy from plant biomass has prompted means to produce oil in vegetative tissues, mostly in leaves. Therefore, we made an attempt to engineer Jatropha plants by overexpressing the *DGAT1* gene that codes for the enzyme responsible for the final and only committed step in TAG biosynthesis, to accumulate TAGs in leaves, in addition to seeds. Hand sections of control and transgenic petioles of Jatropha were examined for determining the intracellular localization of the TAG. Fresh samples from the fifth leaf from apical bud of wild-type and transgenic Jatropha plants were stained with lipid-specific fluorescent dye, Nile red, and observed under a confocal laser scanning microscope after excitation at 559 nm of light. The frequency of oil droplets was found increased in transgenic samples compared to the wild type, and oil droplets mostly distributed close to inner peripheral region of cells (Fig. 5A, B).

### Effect of enhanced TAG accumulation on agronomic traits in transgenic Jatropha lines

**Fig. 6:**
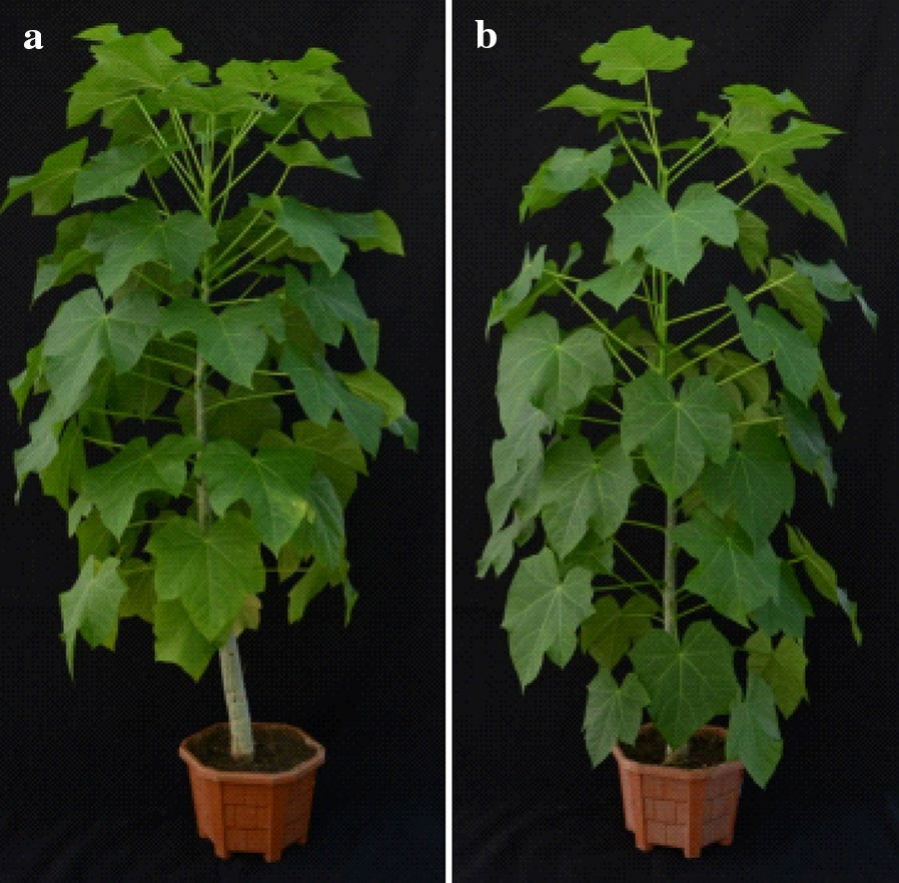
Growth comparison of AtDGAT1 transgenic line and wild-type *Jatropha curcas*. **a** AtDGAT1 transgenic *J. curcas*; **b** wild type. Transgenic plants and wild-type Jatropha plants were planted in pots containing soil, manure, and sand in 1:1:1 ratio. Green house condition was maintained at 25 ± 2 °C, relative humidity 60 ± 5 %, and 16-h photoperiod. The light intensity was maintained at a photosynthetic photon flux density (PPFD) of 240 μM/m^2^/s provided by 40-W cool white fluorescent lamps

We investigated whether there were any negative effects of TAG accumulation on agronomic traits in ATDGAT1 overexpressing Jatropha lines under greenhouse conditions. Five transgenic lines with high TAG content and control WT plants were monitored for agronomic traits including time duration for flowering, plant height, number of primary branches after one trimming, secondary branches, seed number per tree after acclimatization in soil, seed length, seed width, seed breadth, and 10-seed weight (Table 1). The transgenic lines were morphologically no different from the wild type (Fig. 6). The transgenic lines as well as the WT plants took approximately two and half years for flowering, and there were no obvious differences in time taken for inflorescence emergence. There was no significant difference in number of primary branches and secondary branches found among the transgenic lines and WT plants; however, some of the transgenic lines had a marked increase in plant height (Table 1). The transgenic lines had an average of 56.88 ± 7.6 seeds per tree with an average seed weight of 7.46 ± 0.03 g, and with increased average seed length, seed width, and seed breadth (Table 1). These data collected from transgenic lines under greenhouse conditions indicated that high TAG accumulation had no negative effects on important agronomic traits in Jatropha.

**Table 1:** Physiological parameter comparison between wild type and *AtDGAT1* transgenic lines

	Plant height (cm)	Primary branch^a^	Secondary branches^b^	Seed number per tree	Seed length (mm)	Seed width (mm)	Seed breadth (mm)	10 seeds weight (g)
WT	169.47	4	4.5 ± 0.6	76	18.35 ± 0.21	10.65 ± 0.15	8.95 ± 0.29	7.13 ± 0.21
TR1	170.68	3	8.6 ± 0.7	47	18.36 ± 0.12	10.71 ± 0.13	9.19 ± 0.20	7.47 ± 0.07
TR2	179.83	3	5.3 ± 2.7	83	18.94 ± 0.16	10.90 ± 0.13	8.94 ± 0.18	7.39 ± 0.06
TR3	163.07	4	4.6 ± 1.2	43	18.38 ± 0.23	10.72 ± 0.17	9.137 ± 0.12	7.52 ± 0.07
TR4	167.34	3	6.0 ± 0.6	46	18.78 ± 0.21	11.39 ± 0.16	8.64 ± 0.23	7.54 ± 0.05
TR5	172.68	3	4.3 ± 0.7	65	18.35 ± 0.14	10.51 ± 0.14	8.83 ± 0.14	7.43 ± 0.11

^a^Number of branches after pruning

^b^Average secondary branches per primary branch (Sample size for measurement was *n* = 10)

## Discussion

Seed-derived triacylglycerol (TAG), a neutral lipid with twice the energy density of cellulose, is the most suitable for generating high energy density desirable biodiesel with one of the simplest and most efficient manufacturing processes [51–57]. Increasing accumulation of TAGs in seeds as well as vegetative tissues is gaining momentum in order to make plant-derived biodiesel production economically more sustainable [38, 57]. Consequently, engineering plants to accumulate TAG in vegetative tissues and raising the TAG content in seeds are becoming the most potential strategy [54, 55, 58]. Since DGAT1 is the only enzyme that is exclusively committed to TAG biosynthesis in Kennedy pathway, direct upregulation of Kennedy pathway through overexpression of the DGAT1 enzyme responsible for the last and only committed step in seed TAG biosynthesis is most promising.

Here, we describe the generation of transgenic Jatropha plants that accumulates high level of TAGs in seeds as well as in leaves by the overexpression of *Arabidopsis DGAT1*. In this work, we used the CaMV35S promoter because of its strong and constitutive nature in regulating transgene expression to enable ectopic overexpression of *AtDGAT1* in leaves as well as seeds. The enhanced TAG accumulation in transgenic Jatropha lines had no penalty on the growth rates, growth patterns, leaf number, and leaf size of plants. Enhanced expression of AtDGAT1 in transgenic Jatropha lines appears to have increased the total lipid content by 1.5- to 2-fold increase in leaves and 20–30 % in seed kernels. In the best transgenic line (TR1), total lipid content was increased by twofold in leaves and 30 % in seed kernels. This increase was accompanied with no significant change in protein content in leaves, but a minor increase in protein content in seed kernels was found in transgenic plants. In contrast to proteins in leaves and seeds, significant increase in carbohydrate content in leaves and marked decrease in carbohydrate content in seeds of transgenic lines were observed. These results suggest that increased accumulation of total sugar may have contributed to reallocation of precursor for enhanced TAG synthesis in transgenic leaves. Carbohydrates were important osmotic solutes in leaves and seeds, and were potentially involved in the carbon source transformation into lipids. Previous studies suggested that carbohydrate content in seed was correlated with seed oil contents [59]. However, the reallocation of precursor of photosynthesis to TAG biosynthesis is more in leaves than seeds of transgenic AtDGAT1 Jatropha lines, and possibly the contribution to TAG biosynthesis in these transgenic lines by leaves is more than seeds. The mechanisms underlying these changes need to be addressed in future investigations.

We found that AtDGAT1 overexpressing transgenic lines contain in seeds an overall increase in TAG and DAG, and lower FFA levels compared with control wild-type Jatropha plants. Therefore, crude oil extracted from AtDGAT1 overexpressing Jatropha lines would be expected to be a better substrate compared to those of wild-type plants for alkaline transesterification in biodiesel production. Seed oil containing very low levels of FFA and moisture is ideal for biodiesel production as alkaline treatment process which is the preferred method for transesterification owing to its shorter reaction time and reduced energy consumption requires crude oil with very low levels of FFA and moisture [60, 61], as a high level of FFA and water can convert transesterification into saponification, leading to easy depletion of catalysts [62, 63]. However, a thorough analysis of alkaline-treated transesterification with crude oils from *AtDGAT1* overexpressing Jatropha lines should be performed in future studies. Overexpression of *AtDGAT1* in Jatropha resulted in increase of oleic acid in leaves by 20–31 % and in seeds by 9–25 %, and decrease of linoleic acid in both leaves and seeds without compromising its agronomic performance. Nevertheless, these high oleic acid level transgenic lines need to be further characterized for extensive analysis of their suitability for diesel engines. We observed distinct oil droplets in the leaf tissues as revealed by Nile red staining. These observations were consistent with increased oil accumulation in the leaves of *AtDGAT1* overexpressing lines. Intriguingly, the presence of oil bodies typically found only in seeds was detected in the vegetative tissue, suggesting that seed-like biosynthetic mechanisms were perhaps ectopically induced. These results are similar to those reported with the overexpression of LEC2 in senescing leaves [58].

However, more importantly, ectopic overexpression of *AtDGAT1* in Jatropha resulted in an increase in oil content, average plant height, seeds per tree, average 100-seed weight, and seed length and breadth. Thus, there was no penalty in 100-seed weight due to the oil content increase, the result being an increase in total oil on a per seed basis of between 20 and 30 % more in the *AtDGAT1* transgenic lines, thus indicating a 20–30 % net overall oil increase when compared with the wild-type plants [64]. Additionally, *AtDGAT1* overexpression lines of Jatropha also exhibited an oil content increase of 1.5- to 2-fold in leaves. This distinct difference between *AtDGAT1* transgenic and wild-type plants with respect to effects on seed weight, seed length and breadth, and plant height is unclear, and suggests a more complex interaction between the traits of oil increase and seed traits than is currently understood.

## Conclusions

In this paper, we demonstrated that upregulation of TAG biosynthesis by ectopic overexpression of *AtDGAT1* in transgenic *J. curcas* plants leads to an enhanced accumulation of TAGs in leaves as well as seeds without compensating plant and seed traits. In principle, accumulation of oils in leaf foliage and seeds provides an opportunity to enhance the energy density of the biomass and thus have significant impact on biofuel production. This is the first report in Jatropha demonstrating enhanced oil accumulation in both seeds and vegetative tissues. These promising results are a first step towards making an economically viable biofuel crop through transgenic approach. Although a lot of future efforts are to be made to look at partitioning substantial carbon into TAG in vegetative tissues in addition to enhanced accumulation in seeds to make it highly sustainable, our results support the basic feasibility of a strategy to redirect carbon partitioning from starch to oil in plant biomass. The resulting seed oil content changes may have commercial significance in terms of increasing oil content for greater productivity.

## Methods

### Plant material and explant preparation for transformation

Seeds were collected from elite lines of *J. curcas* (IITG JC-19) and maintained in shade house of Indian Institute of Technology Guwahati [65]. The seeds were decoated and soaked in distilled water overnight at room temperature. The soaked decoated seeds were treated for 10 min with 0.1 % sodium hypochlorite solution containing 4–5 drops of Tween-20, followed by washing with distilled water for 20 min. The decoated seeds were then surface sterilized with 0.2 % mercuric chloride for 2 min and finally rinsed with sterile distilled water for 4–5 times. After blot drying on sterilized filter paper, the endosperm was dissected out carefully to expose embryos with papery cotyledonary leaves and germinated on Murashige and Skoog (MS) basal media [66]. The 4-day-old papery cotyledonary leaves were cut into four segments (8 mm^2^) with the edges removed and used as explants for *Agrobacterium*-mediated transformation.

### Construction of *DGAT1* expression construct

Full-length *Arabidopsis thaliana diacylglycerol acyltransferase 1* (DGAT1) (Gene Bank: AF051849.1) cDNA was PCR-amplified with *XbaI* and *BamHI* sites on the 5′- and 3′- ends, respectively, using the forward primer (GCA TCT AGA ATG GCG ATT TTG GAT TC) and reverse primer (GCA GGA TCC TGA CAT CGA TCC TTT TC), and the PCR product was cloned as *XbaI*–*BamHI* fragment and maintained in the intermediate vector, pTZ57R/T. The PCR fragment was inserted into the *XbaI/BamHI* sites of plant expression vector, pBI121 as C-terminal fusion to gus gene under the control of CaMV35S promoter and NOS terminator. The AtDGAT1 construct was mobilized into the disarmed *Agrobacterium tumefaciens* strain EHA105 and used for the transformation.

### Jatropha transformation

Plant transformation of Jatropha was carried out using the protocol described earlier by our lab [40]. In brief, the protocol consisted of four steps: co-cultivation, shoot induction, shoot elongation, and rooting. The explants after inoculation with *Agrobacterium* suspension were co-cultivated for 3 days. After co-cultivation, the explants were transferred to callus induction medium (CI, MS medium supplemented with 6.66 µM BAP and 0.24 µM IBA) containing 500 mg/L cefotaxime and 400 mg/L augmentin in dark condition. The cultures were transferred to fresh CI medium at an interval of 5, 7, and 8 days. After 3 weeks of culture, the calli were transferred onto shoot regeneration medium (SR, MS medium supplemented with 6.66 µM BAP, 0.24 µM IBA, 1.44 µM GA_3_) containing 50 mg/L kanamycin, 500 mg/L cefotaxime, and 400 mg/L augmentin, and incubated at 16 h photoperiod. The cultures were periodically transferred onto fresh selection medium. After 4 weeks of culture on selection, the regenerating kanamycin-resistant shoots were detached and transferred to shoot elongation medium (SE, MS medium supplemented with 1.0 µM GA_3_) containing 15 mg/L kanamycin and 400 mg/L augmentin. After a week, the elongated shoots were shifted to root induction medium (RI, ½ MS medium supplemented with 0.5 µM IBA) and 400 mg/L augmentin. Well-rooted transformed plantlets were washed thoroughly in running tap water, and acclimatized and maintained in greenhouse as per our lab protocol described earlier [65].

### Molecular analysis of transgenic Jatropha plants

#### Polymerase chain reaction (PCR) analysis

Genomic DNA was isolated from the untransformed (UT) and putative transformed Jatropha plants using the NucleoSpin Plant II Maxi kit (Macherey–Nagel, Duren, Germany). PCR was carried out to detect the presence of *AtDGAT1*, *nptII,* and *gus* in transformed Jatropha plants. The 384-bp region internal to *AtDGAT1*, 540-bp region internal to *nptII,* and 400-bp region internal to *gus* were amplified using primers (*AtDGAT1Fw*: TCT GCT GGC GTT ACT ACG GT and *AtDGAT1*Rv: CGG CAT GGC TCT GTT TGA AG; *nptII*Fw: GTG GAG AGG CTA TTC GGC TA and *nptII*Rv: CCA CCA TGA TAT TCG GCA AC; and *gus*Fw: GGT GGG AAA GCG CGT TAC AAG and *gus*Rv: TGG ATT CCG GCA TAG TTA AA) using the PCR conditions: 95 °C for 5 min (1 cycle), 95 °C for 1 min, 58 °C for 1 min, 72 °C for 1 min for 35 cycles followed by a final extension of 72 °C for 5 min. The recombinant plasmid pBI21*AtDGAT1* was used as positive control. The PCR-amplified products were analyzed on 1 % agarose gel and visualized by ethidium bromide staining.

#### RNA isolation and semi-quantitative RT-PCR analysis

The total RNA was isolated from the PCR-positive transgenic Jatropha lines and wild-type untransformed plants using RNA extraction kit (NucleoSpin RNA Plant, MN, Germany) and quantified with nanodrop spectrometer (Nanodrop, USA). The cDNA was prepared using 1 µg of total RNA using reverse transcription kit (ThermoScientific, USA), according to its manufacturer’s instructions. The semi-quantitative RT-PCR was performed using primers (*AtDGAT1*Fw: GGTGGCGGAGAGTTCGTCGA and *AtDGAT1*Rv: TCTTCCTTCTCCGCCGCCTC; *JcActin*Fw: ATGAGCTTCGAGTTGCAC and *JcActin*Rv: ACCATCACCAGAATCCAG) for amplifying a 249-bp fragment of *AtDGAT1* and a 590-bp of *JcActin* as an internal control to indicate the amount of starting RNA. Semi-quantitative PCR was amplified, and amplification products were analyzed with 1 % agarose gel, detected by ethidium bromide staining.

### Southern blot analysis

Randomly selected PCR-positive T_0_ transgenic Jatropha lines were analyzed by Southern hybridization for the integration of transgene. Genomic DNA was isolated from leaves of transgenic and control untransformed (UT) plants using the NucleoSpin Plant II Maxi kit (Macherey–Nagel, Duren, Germany). Sixty microgram of genomic DNA was digested with *BamHI* and separated on a 0.8 % agarose gel. The gel was processed and transferred to ZetaProbe nylon positively charged membrane (Bio-Rad, USA) following standard procedures. The blot was hybridized with DIG-labeled 0.54-kb *nptII* PCR-amplified product as probe. Hybridization and detection of signals were carried according to the DIG Labeling and detection supplier instructions (Roche Diagnostics, Mannheim, Germany).

### Stable *GUS* assay

Histochemical GUS assays were used to assess the stable GUS expression [67] in leaf explants after three days of co-cultivation, callus, stem, leaf, roots, and seeds of transgenic plants.

### Fatty acid analysis in transgenic Jatropha

Total lipids were isolated following the method described by Bligh and Dyer [68]. Two hundred milligram of leaves and 500 mg seeds kernel of control untransformed and transgenic lines were homogenized in mortar-pestle, and lipids were extracted from organic phase. Lipid fraction in bottom phase was collected in glass tube and evaporated in rotary evaporator. Total lipids were quantified after drying in a desiccator for 24 h. The weight of the total oil was determined gravimetrically, and oil content was recorded as the ratio of lipid and oil to dried leaf sample and seed kernel weight.

### Analysis of lipids by thin-layer chromatography

Lipids were fractioned from neutral lipids by thin-layer chromatography. On silica gel plate (TLC Silica Gel 60 F_254_, Merck), 150 µg of extracted lipid was spotted and resolved using solvent system of Hexane: diethyl ether: acetic acid (70:30:1, v/v/v). Triacylglycerol spots were revealed by staining with iodine vapor.

### Fatty acid methyl ester (FAME) preparation and analysis by Gas chromatography

We used GC to analyze the FA profile of transgenic Jatropha lines. About 20 mg of lipid was dissolved in methanol in a test tube, and 0.5 M potassium hydroxide in anhydrous methanol was added with reaction volume of 20 mL. The solution was maintained at 60 °C for 30 min. The methyl esters were extracted with hexane (2 × 5 mL), and the organic phase was washed twice with Milli-Q water to remove any aqueous impurities. Organic phase was collected in screw cap glass tube, and solvent was removed in rotary evaporator; after filtration through a 0.2-μm filter, methyl esters were used for GC analysis. FAME analysis was performed on Varian 450-GC (Varian Capillary Column CP-SiL8 CB, 30 m 0.25-mm i.d., 0.25-µm film thickness). FAMEs were separated and detected by flame ionization detector (FID). Nitrogen was used as carrier gas with 0.4 ml s^−1^ at constant flow rate. The oven regime: 140 °C for 5 min, 180–240 °C at 3 °C/min, and hold at 220 °C for 10 min. The injector and detector temperatures were kept at 250 °C, and 1 µL injection volume at split ratio of 1:20 was used for the analysis. Peaks were identified based on their retention times compared with a FAME reference mixture (Supelco, Bellenfonte, PA, USA). Fatty acid composition was calculated based on the peak area percentage of total fatty acids.

### Protein and carbohydrate analysis

Protein content of Jatropha transgenic lines was determined as described by Focks and Benning [69] using 200 mg of each leaf and dried endosperm. Protein amounts were measured according to Bradford [70] using three technical replications using ready to use Bradford’s reagent (Fermentas, USA). To analyze the carbohydrate content, 200 mg of each of leaf and dried endosperm were homogenized in 200 μL of assay buffer and centrifuged at full speed. The extracted supernatant was used for quantification of carbohydrate using a Total Carbohydrate Assay Kit (Sigma-Aldrich). d-glucose was used as a standard for calibration, and data were expressed as mg carbohydrate g^−1^ tissue fresh weight.

### Seed weight and size determination

Mature seeds were harvested from untransformed control (UT) and transgenic Jatropha lines grown under the same conditions. The seeds were then weighed carefully on analytical balance with sample size 50; values (*n* = 5) are given as mean ± SD. LIA image processing software (Nagoya University, Japan) was used to measure seed sizes. Values (*n* = 10) are given as mean ± SD. The moisture content was determined as the difference between the initial and dry weights divided by the initial seed weight and represented in percentage.

### Microscopy analysis

Leaf sections of transgenic and control Jatropha plants were stained with Nile red (HiMedia, India), mounted in 70 % glycerol, and visualized using a laser confocal scanning microscope. Oil droplets were observed at 570–630 nm emission following 559-nm excitation by solid-state laser. Images were captured with LSM 510 META laser scanning microscope (Lieca, Germany).

## Abbreviations

TAG: triacylglycerol
DAG: diacylglycerol
DGAT: diacylglycerol acyltransferase
FFA: free fatty acid
FAME: fatty acid methyl esters
BAP: 6-benzylaminopurine
IBA: indole-3-butyric acid
GA3: gibberellic acid
GC: gas chromatography
PCR: polymerase chain reaction
bp: base pairs
